# Horn, Eva. 2024. *Klima: Eine Wahrnehmungsgeschichte*. Frankfurt a. M.: S. Fisher. Livingstone, David N. 2024. *The Empire of Climate: A History of an Idea*. Princeton, NJ: Princeton University Press

**DOI:** 10.1177/14744740251386837

**Published:** 2025-10-15

**Authors:** Maximilian Gregor Hepach

**Affiliations:** Department of Geography, Durham University, Lower Mountjoy, South Road, Durham DH1 3LE, UK

Looking towards the uncomfortably near climate future, apocalyptic scenarios of irreversible tipping-points intensify the public recognition of how deeply society and culture are connected to climate. Two recent monographs on the history of climate as an idea (David Livingstone) and experience (Eva Horn) invite cultural geographers to revisit this complicated connection at a pressing moment, touching upon two tenets of physical and human geography in the process.

On the one hand, climate change has long been scientifically defined as something that takes place on scales of place and time that escape immediate experience. At present, however, extreme weather events are routinely viewed and reported on as evidence for (or even experiences of) climate change. How we draw the line between weather and climate is changing.^1^ On the other hand, emphasising our individual and collective embeddedness in a local and global climate resonates uneasily with the history of human geography as a discipline, namely the legacy of environmental determinism. Consequently, recent human and cultural geography has been less interested in climate as a fact of nature. Instead, work has focussed on the multiplicity of ‘climate’, e.g. how climate is culturally constructed, politically contested, or revisited through the critical prism of work in feminist new materialism as well as Indigenous and Black studies differently.^2^

David Livingstone’s *The Empire of Climate: A History of an Idea* provides a wealth of evidence to confirm the suspicion that theories of climate are complicit in furthering environmental determinism. He draws on the long history of climate as an idea to situate our present moment within recurring motifs of health, mind, wealth, and war; motifs which structure the four parts of the book. With the benefit of hindsight, historical scientific theories of the relation between weather, climate, and, say, health are clearly saturated with the racial, colonial, and imperial politics of their time; laden with ‘moral cargo’ (p. 406). Viewing present-day debates around weather- and climate-health as part of this history helps caution and sensitise cultural geographers to the pitfalls of assigning climate too great a role now. In ‘climate determinism’, Livingstone identifies ‘the flip side of human-induced climate change, the alter ego of the Anthropocene’ (p. 400).

Livingstone’s expansive discussion of material across various time periods, geographic places, and scientific disciplines exemplifies how climate functions as a sort of ‘empty’ or ‘floating signifier’.^3^ Climate does not denote a singular object but signifies a whole constellation of meaning subject to change.^4^ What remains consistent and becomes a sort of historical necessity, in Livingstone’s account, is how any given constellation of meaning signified *by* climate becomes reified *in* climate. What is culturally constructed becomes environmentally determined, e.g. racial prejudice becomes race determined by climate. For cultural geography, recognising climate as a floating signifier entails a sustained awareness of the wider constellation of meaning climate represents. This task is easier the further the historical distance to the material is. Of greater urgency and difficulty is applying this approach to present-day climate change discourse.

As the subtitle to *The Empire of Climate* suggests, Livingstone’s interest lies in the *idea* of climate, the meanings climate holds. The subtitle of Eva Horn’s *Climate: A Perceptual History* indicates a different interest, namely in perception and experience. Revisiting scientific and cultural history, Horn elaborates the aesthetic, scientific, political, and philosophical possibilities of being-in-climate. It is not the similarities and affinities through which Horn renders her historical examples persuasive. This would be the case in a more genealogical approach (Livingstone). Instead, Horn foregrounds the differences and contradictions between modern-day and past climate concepts, e.g. in 19^th^ century climate anthropologies (Montesquieu; Herder) and present-day climate science. Different historical concepts and related experiences do not directly translate into one another, but rather ‘resonate’ across differences.^5^ The shared transmitter and receiver of these resonating frequencies is not climate as an idea, but the phenomenological subject. Consequently, the material Horn draws upon is not only theoretical, philosophical, and scientific work, but is also informed by literature (e.g. Thomas Mann), art (e.g. J. M. W. Turner), and architecture (e.g. Philippe Rahm), in which atmospheric experience finds more immediate expression.

A key contribution of Horn’s, in this respect, is the way perceptual history is mobilised not to construct a comprehensive definition of climate — a definition which might turn imperial and reify a constellation of ideas — but rather to intensify climate experience (p. 21). Horn self-consciously positions her work as a response to a scientific concept of climate that denies experience (p. 16), adding to the possibilities of climate as an idea.^6^ Whereas Livingstone is cautionary about the overzealous use of climate, underlying Horn’s arguments is a latent optimism: recognising how our shared belonging-to-air [Luftverbundenheit] might yield a basis for a just and sustainable politics (p. 497).^7^

Livingstone’s and Horn’s work provides expansive as well as rich empirical and conceptual material, evidencing both the breadth of climate’s actual and imagined influence on culture and the different media in which this influence comes to be represented: scientific theories, philosophical treatises, medical advice, modern and classical works of literature, artworks, articles of clothing, and more. Attention to this multitude presents a variety of starting points for cultural geographers to expand upon.

Returning to the question of determinism, scientific and public concern around the impact of climate change on human minds and bodies is increasing.^8^ Cultural geographers might be advised to consider the nature of this influence critically but seriously, as some work engaging with the elemental and critical geographies of heat, for example, already does.^9^ With Livingstone’s cautions in mind, Horn’s nuanced account of the modulation or attunement of human bodies (and minds) through and in resonance with their environment might serve as an example of how to thread the needle of climate’s enmeshment with culture (p. 123-124). In view of cultural geography’s rich intellectual legacy — questioning assumptions of presence, representation, and the privileging of meaning over matter — both works further invite the reader to reconsider the nature of climate and its influence.

## Footnotes

1 C. Colebrook, ‘A Globe of One’s Own: In Praise of the Flat Earth,’ *SubStance* XLI (2012), pp. 30–39.

2 M. Mahony, ‘Climate Change and the Geographies of Objectivity: the Case of the IPCC’s Burning Embers Diagram,’ *Transactions (Institute of British Geographers: 1965)* XL (2015), pp. 153–167; M. Hulme, *Weathered: Cultures of Climate* (London, SAGE, 2016); H. Bulkeley, ‘Navigating Climate’s Human Geographies: Exploring the Whereabouts of Climate Politics,’ *Dialogues in Human Geography* IX (2019), pp. 3–17; S. Engelmann and D. McCormack, ‘Elemental Worlds: Specificities, Exposures, Alchemies,’ *Progress in Human Geography* XLV (2021), pp. 1419–1439; S. Wright and M. Tofa, ‘Weather Geographies: Talking about the Weather, Considering Diverse Sovereignties,’ *Progress in Human Geography* XLV (2021), pp. 1126–1146.

3 M. Müller, ‘Doing Discourse Analysis in Critical Geopolitics,’ *L’Espace Politique* XII (2011).

4 On climate as an idea, see M. Hulme, ‘Climate. Living Lexicon for the Environmental Humanities,’ *Environmental Humanities* VI (2015), pp. 175–178; H. Knox, *Thinking Like a Climate* (Durham, NC, Duke University Press, 2020).

5 W. C. Dimock, ‘A Theory of Resonance,’ *Publications of the Modern Language Association of America* CXII (1997), pp. 1060-1071.

6 See also B. Schneider, *Klimabilder: eine Genealogie globaler Bildpolitiken von Klima und Klimawandel* (Berlin, Matthes & Seitz, 2018).

7 On the geographies of ‘belonging to air’, see P. Adey, ‘Air’s Affinities: Geopolitics, Chemical Affect and the Force of the Elemental,’ *Dialogues in Human Geography* V (2015), pp. 54–75; M. Nieuwenhuis, ‘Breathing Materiality: Aerial Violence at a Time of Atmospheric Politics,’ *Critical Studies on Terrorism* IX (2016), pp. 499–521; S. Engelmann, *Sensing Art in the Atmosphere: Elemental Lures and Aerosolar Practices* (London, Routledge, 2020).

8 One notable example is C. Aldern, *The Weight of Nature* (London, Penguin, 2024).

9 G. Adams-Hutcheson, ‘Farming in the Troposphere: Drawing Together Affective Atmospheres and Elemental Geographies,’ *Social & Cultural Geography* XX (2019), pp. 1004–1023; C. Robinson, ‘Raising the Temperature: A Critical Geographical Perspective on Heat,’ *Progress in Environmental Geography* IV (2025), pp. 165–189.

